# Brain-Computer Interfaces: Towards Practical Implementations and Potential Applications

**DOI:** 10.1155/2007/62637

**Published:** 2008-02-12

**Authors:** F. Babiloni, A. Cichocki, S. Gao

**Affiliations:** ^1^Dipartimento di Fisiologia Umana e Farmacologia “Vittorio Erspamer”, Università delgi Studi di Roma “La Sapienza”, Piazzale Aldo Moro 5, 00185 Roma, Italy; ^2^Laboratory for Advanced Brain Signal Processing (ABSP), RIKEN Brain Science Institute, 2-1 Hirosawa, Wako-shi, Saitama 351-0198, Japan; ^3^Department of Biomedical Engineering, Tsinghua University, Beijing 100084, China

Brain-computer interfaces (BCIs) are systems that use brain signals (electric, magnetic, metabolic) to control external devices such as computers, switches, wheelchairs, or neuroprosthesis. While BCI research hopes to create new communication channels for disabled or elderly persons using their brain signals, recently efforts have been focused on developing potential applications in rehabilitation, multimedia communication, and relaxation (such as immersive virtual reality control). The various BCI systems use different methods to extract the user’s intentions from her/his brain electrical activity. Many researchers world wide are actually investigating and testing several promising BCI paradigms, including (i) measuring the brain activities over the primary motor cortex that results from imaginary limbs and tongue movements, (ii) detecting the presence of EEG periodic waveforms, called steady-state visual evoked potentials (SSVEPs), elicited by flashing light sources (e.g., LEDs or phase-reversing checkerboards), and (iii) identifying event-related potentials (ERPs) in EEG that follow an event noticed by the user (or his/her intention), for example, P300 peak waveforms after a target/rare (oddball) stimulus among a sequence the user pay attention to. One promising extension of BCI is to incorporate various neurofeedbacks to train subjects to modulate EEG brain patterns and parameters such as event-related potentials (ERPs), event-related desynchronization (ERD), sensorimotor rhythm (SMR), or slow cortical potentials (SCPs) to meet a specific criterion or to learn self-regulation skills. The subject then changes their brain patterns in response to some feedbacks. Such integration of neurofeedback in BCI is an emerging technology for rehabilitation, but we believe it is also a new paradigm in neuroscience that might reveal previously unknown brain activities associated with behavior or self-regulated mental states. The possibility of automatic context-awareness as a new interface goes far beyond the standard BCI with simple feedback control. BCI relies increasingly on findings from other disciplines, especially, neuroscience, information technology, biomedical engineering, machine learning, and clinical rehabilitation.

This special issue covers the following topics:

- noninvasive BCI systems (EEG, MEG, fMRI) for decoding and classification neural activity in humans;
- comparisons of linear versus nonlinear signal processing for decoding and classifying neural activity;
- multimodal neural imaging methods for BCI;
- systems for monitoring brain mental states to enable cognitive user interfaces;
- online and offline algorithms for decoding brain activity;
- signal processing and machine learning methods for handling artifacts and noise in BCI systems;
- neurofeedback and BCI;
- applications of BCI, especially, in therapy and rehabilitation;
- new technologies for BCI, especially, multielectrode technologies interfacing, telemetry, wireless communication for BCI.

This special issue includes 23 contributions which cover a wide range of techniques and approaches for BCI and related problems.

